# In order to lower the antinutritional activity of serine protease inhibitors, we need to understand their role in seed development

**DOI:** 10.3389/fpls.2023.1252223

**Published:** 2023-10-04

**Authors:** Juan Vorster, Willem van der Westhuizen, Gedion du Plessis, Diana Marais, Francesca Sparvoli, Eleonora Cominelli, Emanuela Camilli, Marika Ferrari, Cinzia Le Donne, Stefania Marconi, Silvia Lisciani, Alessia Losa, Tea Sala, Karl Kunert

**Affiliations:** ^1^ Department Plant and Soil Sciences, Forestry and Agricultural Biotechnology Institute, University of Pretoria, Pretoria, South Africa; ^2^ National Research Council, Institute of Agricultural Biology and Biotechnology (CNR-IBBA), Milan, Italy; ^3^ Council for Agricultural Research and Economics, Research Centre for Food and Nutrition, Rome, Italy; ^4^ Council for Research in Agriculture and Economics, Research Centre for Genomics and Bioinformatics, Montanaso Lombardo, Italy

**Keywords:** serine proteases, serine protease inhibitors, seed development, seed viability, antinutrients, abiotic stress

## Abstract

Proteases, including serine proteases, are involved in the entire life cycle of plants. Proteases are controlled by protease inhibitors (PI) to limit any uncontrolled or harmful protease activity. The role of PIs in biotic and abiotic stress tolerance is well documented, however their role in various other plant processes has not been fully elucidated. Seed development is one such area that lack detailed work on the function of PIs despite the fact that this is a key process in the life cycle of the plant. Serine protease inhibitors (SPI) such as the Bowman-Birk inhibitors and Kunitz-type inhibitors, are abundant in legume seeds and act as antinutrients in humans and animals. Their role in seed development is not fully understood and present an interesting research target. Whether lowering the levels and activity of PIs, in order to lower the anti-nutrient levels in seed will affect the development of viable seed, remains an important question. Studies on the function of SPI in seed development are therefore required. In this Perspective paper, we provide an overview on the current knowledge of seed storage proteins, their degradation as well as on the serine protease-SPI system in seeds and what is known about the consequences when this system is modified. We discuss areas that require investigation. This includes the identification of seed specific SPIs; screening of germplasms, to identify plants with low seed inhibitor content, establishing serine protease-SPI ratios and lastly a focus on molecular techniques that can be used to modify seed SPI activity.

## 1 Introduction

Proteases are involved in the entire life cycle of plants with cysteine proteases the most abundant and investigated followed by serine proteases and aspartic proteases. The model plant *Arabidopsis thaliana* for example has approximately 700 genes coding for proteases (Rawlings et al., 2018; Marshall and Vierstra, 2019). Plant proteases act as regulators of physiological processes including protein processing and homeostasis, organelle development, seed germination, environmental stress response as well as senescence and programmed cell death. Uncontrolled protease activity is, however, harmful for plant growth. Regulation of this protease activity by intracellular protease inhibitors is, therefore, of utmost importance (Rachel and Sirisha, 2014; Sharma and Gayen, 2021). Proteases inhibitors are grouped into families, classified based on the specific reactive sites present. These are cysteine-, metalloid- aspartic and serine protease inhibitors. In plants they can also be classified according to their specific structural or biochemical properties such as the Bowman-Birk (BBI) and Kunitz type serine protease inhibitors (KTI). (Clemente et al., 2019). Protease inhibition occurs based on one of two mechanisms, irreversible trapping that is irreversible or a tight binding interaction where proteases and inhibitors co-exist in a stable equilibrium. SPI falls within the latter group. In addition to the regulation of the activity of exogenous proteases, derived, for example, from insect pests and pathogens feeding or attacking plants, these inhibitors are important in plant cellular homeostasis and survival (van der Hoorn and Rivas, 2018). Serine protease inhibitors (SPIs), such as KTIs, and the much smaller BBIs, can additionally act as antinutrients in humans and animals. The quantity of these inhibitors in seeds varies depending on plant species and variety (Fernandes et al., 1991). In mature seeds of the marama bean, trypsin inhibitors can represent up to 10% of the total seed protein amount (Elfant et al., 1985; Cullis et al., 2023).

Unfortunately, the exact biological function of SPIs has not yet been fully elucidated (Grosse-Holz and van der Hoorn, 2016). This is despite the fact that SPIs are present in different organs and associated with different functions, such as defense against biotic and abiotic stresses (Sun et al., 2015; Rodríguez-Sifuentes et al., 2020; Mangena, 2022). In this regard, any detailed work is still scarce on demonstrating a particular function of SPIs in viable seed production. In this paper, we will outline the reasons we believe studies on the function of SPIs in seed are urgently required, particularly when the aim is to decrease the anti-nutrient activity of these PIs in seeds. We, therefore, provide an overview of seed storage proteins and their degradation, an overview of the current knowledge regarding the role of the serine protease – SPI system in seed and the known consequences of attempting to modify this system. We then give an outline of areas, that require substantial research before lowering SPI activity in seeds, for lowered anti-nutrient activity to be achieved.

## 2 Seed storage proteins and their degradation

Storage proteins make up 80% of the total protein in seed and can be targets of proteases. These proteins are produced during seed development and maturation. In dicot plant species, storage proteins are located in the mesophyll of cotyledons as well as in the embryonic axis (Schlereth et al., 2000). In contrast, monocot plants, such as cereals, have grains which are endospermic seeds. Grains have, however, much less protein than legume seeds (Wakasa and Takaiwa, 2013). Globally wheat *(Triticum aestivum*) is one of the three most important grain crops and cultivated for its importance as a staple food and protein source. Wheat seeds mainly consist of the embryo and the endosperm. Both facilitate seed germination as well as subsequent plant growth and development. The wheat embryo develops from the oosperm and holds a large amount of sugar, fats and proteins that represents 2.8 – 3.5% of the total seed weight (Liu et al, 2021a)

Storage proteins accumulate mainly in the endosperm tissue (Shewry and Halford, 2002) and stored proteins represent 10–12% of the total seed dry weight. There are four categories of seed storage protein which include albumins and globulins (dicot storage proteins) as well as glutelins and prolamins (monocot storage proteins) (Radhika and Rao, 2015). During seed germination, the embryo secretes several enzymes to degrade the storage proteins in the endosperm, which develops from the nucleus after fertilization, to provide nutrition for growth. The endosperm contains mainly storage proteins and starch in addition to some fat and mineral elements. These components ultimately provide energy and raw materials for seed germination (Liu et al., 2021a).

Storage proteins accumulate in protein storage vacuoles and or protein bodies (Pedrazzini et al., 2016). Following germination, storage proteins are degraded with the help of proteases to form free amino acids. These are required for the synthesis of new proteins as well as other nitrogen-containing compounds in the seedling (Liu et al., 2021b). Proteolysis, which is vital for life, is generally required in all organisms for protein turnover by non-selective protein degradation. Proteolysis of seed storage proteins, due to protease action, is, therefore, an important process during seed germination. In cereals as well as legumes, these proteases are highly expressed during germination (Diaz-Mendoza et al., 2019; Szewińska et al., 2016). Based on protein annotations, 6% of the known proteases is associated with seed germination or embryo development (Escandón et al., 2022). Several protease families are involved in the germination process that include cysteine, serine, threonine, aspartic as well as metallo-proteases (Diaz-Mendoza et al., 2016; Rustgi et al., 2018). The catalytic residues of the active sites differ between families with cysteine proteases containing a Cys–His–Asn triad at the active site, serine proteases a His-Asp-Ser triad, while threonine proteases have an active site with a threonine residue at the N-terminal. Aspartic proteases finally possess a dyad of two aspartates and the metallo-proteases usually have a Zn^2+^ in their active site (Rustgi et al., 2018). Most proteolytic enzymes involved in the degradation of seed storage proteins during germination are cysteine proteases with serine, aspartic and metalloproteases also being involved (Tan-Wilson and Wilson, 2012). Protein breakdown is however regulated by endogenous PIs with specific activity towards the various proteases. These inhibitors are, therefore, also constitutive components of seeds and storage organs (Escandón et al., 2022). Due to their abundance in many seeds, the seed PIs are probably playing a twofold function. Firstly, they function as protection against proteases from other non-plant organisms, for example insects and pathogens, and secondly the regulation of endogenous proteases during mobilization of reserve proteins (Hartl et al., 2011; van der Hoorn and Rivas, 2018). However, the precise function of the protease-protease inhibitor system in the regulation of seed protein accumulation and composition as well as seed germination is still not fully characterized.

## 3 Serine protease-serine PI system

Serine proteases affect, alongside cysteine proteases of papain family (C1A) and the legumain family (C13), all stages of the plant life cycle (Schaller et al., 2018). The members of the S10 serine carboxypeptidases (SCP) have particularly been implicated in the cereal seed germination process (Diaz-Mendoza et al., 2016; Martinez et al., 2019). However, in contrast to cysteine proteases, relatively little is currently known about the involvement of serine proteases in the development of viable seeds. Most knowledge so far gained is from investigating cereal seeds. Members of the S10 serine carboxypeptidases are involved in monocot cereal grain germination (Tan-Wilson and Wilson, 2012) and cowpea germination (Lima et al., 2019; Drzymała et al. 2012) reported that serine carboxypeptidases I and III from triticale grains are involved in the degradation of seed storage proteins that were proteolytically modified by a cathepsin L-like proteases. Upon hormonal induction, the rice SCP46 serine carboxypeptidase also regulates grain filling and seed germination (Li et al., 2016). In Vicia seeds, subtilisin-like (S8) serine proteases further participate, alongside cysteine proteases, in the breakdown and mobilization of seed reserve proteins (Schlereth et al., 2000). A serine protease from soybean seedling cotyledons also initiates the proteolysis of the β-conglycinin storage proteins (Morita et al., 1994).

Serine PIs that control serine protease activity are ubiquitously present in many plant species. They have also diverse biological functions and can play dual roles with their physiological functions conserved and their functions diversified by a positive selection pressure (Sin et al., 2006). Little investigated has been, however, the existence of seed specific inhibitor genes. Although many functions of SPIs are not well clarified, researches have suggested that these inhibitors control exogenous proteolytic activity, for example in the defense against herbivores. They possibly also function in plant metabolism and development by regulating endogenous serine protease activity during seed germination and development as well in the mobilization of storage proteins (Roberts et al., 2003; Hartl et al., 2011; Clemente et al., 2019). Importantly, serine proteases, in contrast to cysteine proteases, are part of the human digestive system. Serine PIs with anti-trypsin activity are able to block serine proteases in the human digestive system. These inhibitors accumulate in high levels in legume seeds and can severely reduce the digestibility of a legume meal. It is, therefore, necessary to inactivate these inhibitors before consumption. Presence of inhibitors greatly affects consumer acceptance of beans which are particularly high in inhibitors with anti-trypsin activity. Decreasing the amount of SPI in legume seeds, are, therefore, a consideration in order to improve their nutritional value (Samtiya et al., 2020).

The two major groups of SPI in legume seeds are the Kunitz-type inhibitors (KTIs) and Bowman-Birk inhibitors (BBIs) (for an overview see: Bonturi et al., 2020; Hellinger and Gruber, 2019; Gitlin-Domagalska et al., 2020; Xie et al., 2021). Protease inhibitors were originally proposed to be storage proteins (Pusztai, 1972), being present in storage tissues such as seeds and tubers (Jørgensen et al., 2011). The number of disulfide linkages is a major difference between the BBIs and KTIs, with BBIs usually containing seven while KTIs only containing two linkages. KTIs have further a single reactive site whereas BBIs have two reaction sites. Legumes contain members of both families. Compared to KTIs, BBIs are particularly abundant in common beans and lentil seeds. Both serine PIs are also relatively heat stable and capable of inhibiting two types of serine proteases, trypsin and chymotrypsin, either independently or simultaneously. Chymotrypsin-like proteases are possibly absent in plants (Hohl et al., 2017).

No major effect on plant development or growth occurred when a SPI was silenced (Hartl et al., 2010). Three Bowman-Birk inhibitors BBI-A, BBI-CII and BBI-DII are expressed during development of soybean seeds, with maximal expression during the intermediate stages of seed development and with decreased expression as seeds mature (de Almeida Barros et al., 2012). The inhibitor SaPI2a from *Solanum americanum*, expressed in ovules and young seeds, is further a strong inhibitor of subtilisin (Sin et al., 2006). The specificity of SnSPI2a, and also SnSPI2b, towards subtilisin suggests that SPIs interact with a plant subtilase in the ovary since silencing these inhibitors results in a defect in seed development (Hartl et al., 2011).

## 4 Modifying the serine protease-serine PI system

Attempts to modify the expression of the components of the protease-protease inhibitor system have predominantly been aimed at improving environmental stress tolerance of plants or to increase the amount of seed storage proteins. Many studies have thereby focused on the roles of proteases and their inhibitors in defense against insect and pathogen attack (Kiggundu et al., 2010; Kidric et al., 2014) and also in coping with abiotic stress (Quain et al., 2014; Kunert et al., 2015). Recent findings indicate that, for example, water deficiency regulates the expression of a Kunitz-type inhibitor in *Trifolium repens* to maintain cellular homeostasis (Islam et al., 2017). Changes in the expression profile of Kunitz-type SPIs in response to water limitation further suggests that these inhibitors specifically target serine protease and modify their activity (Kidric et al., 2014; D'Ippólito et al., 2021).

Lowering seed protease activity has also been a research target aimed to increase the seed protein amount (Quain et al., 2014; Kunert et al., 2015). Still, relatively little is known about the involvement of SPIs in viable seed development. A possible indication that SPIs indeed play an important role in seed development has been so far derived from studies with tobacco and other Solanum species. Transgenic tobacco plants constitutively expressing a SPI under the 35S promoter had enhanced seed germination, increased root length with a higher root-shoot ratio as well as a significantly higher total chlorophyll content and also lowered thiobarbituric acid-reactive substances (Srinivasan et al., 2009). A study by Malefo et al. (2020), investigating the role of a BBI in transgenic Arabidopsis under drought stress, found elevated drought tolerance in transgenic plants associated with a reduction in drought-induced oxidative stress. Unfortunately, this study did not investigate the effect of overexpression of the inhibitor on the development of viable seeds, but our group is currently investigating seed development in BBI overexpressing Arabidopsis lines. Knock-down RNAi lines for specific members of the KPI gene family had further increased proline accumulation under well-watered as well as under water deficit conditions as well as modified expression of ethylene biosynthesis genes (Islam et al., 2017). These results suggest that the KPI family has various *in planta* protease target processes which might include regulating proteases during germination but also regulating the defense-response.

There still remains a large gap in our knowledge with regard to the effects of lowering the PI content or activity on seed development and germination. A study by Clemente et al. (2015) tested so far available germplasm resources together with TILLING with the specific aim of lowering the antinutrient content of pea seeds by reduction of the protease inhibitor amounts. In a previous study on soybean, a transgenic line expressing a mutant BBI, in which both active sites were disrupted by the insertion of a glycine residue, showed that seeds had a significantly decreased inhibitor activity (Livingstone et al., 2007). In a soybean accession (PI 157740) with a frameshift mutation in the Kunitz-type KTI3 (Gm08g341500) gene, KTI mRNA accumulation was blocked in embryos during seed development (Jofuku et al., 1989). Transgenic KTI soybean plants, carrying *kti1* and *kti3* mutations, also had dramatically reduced (∼40%) trypsin inhibitor activity (Jofuku et al., 1989; Gillman et al., 2015). A pea line lacking pea albumin2, lectin and two major trypsin inhibitor genes showed improved seed protein digestibility and amino acid content without affecting yield or seed protein concentration (Olías et al., 2023). However, a more detailed characterization of these mutants, regarding their effect on seed development and germination, is still lacking.

Finally, in feeding studies animals fed on a protein meal with lower KTI3 levels had a much better weight gain compared when fed with a raw soybean meal with functional KTI3 (Perez-Maldonado et al., 2003). But feeding with mutant lines was still inferior to feeding with heat-treated soybeans conventionally used to eliminate KTI activity. Unfortunately, lines low in trypsin inhibitor activity are more susceptible to pathogens. Knockout of the BBI *APIP4* in rice enhanced, for example, the susceptibility to the fungal pathogen *Magnaporthe oryzae* (Zhang et al., 2020).

## 5 Areas for intensive future exploration

**Figure 1:**
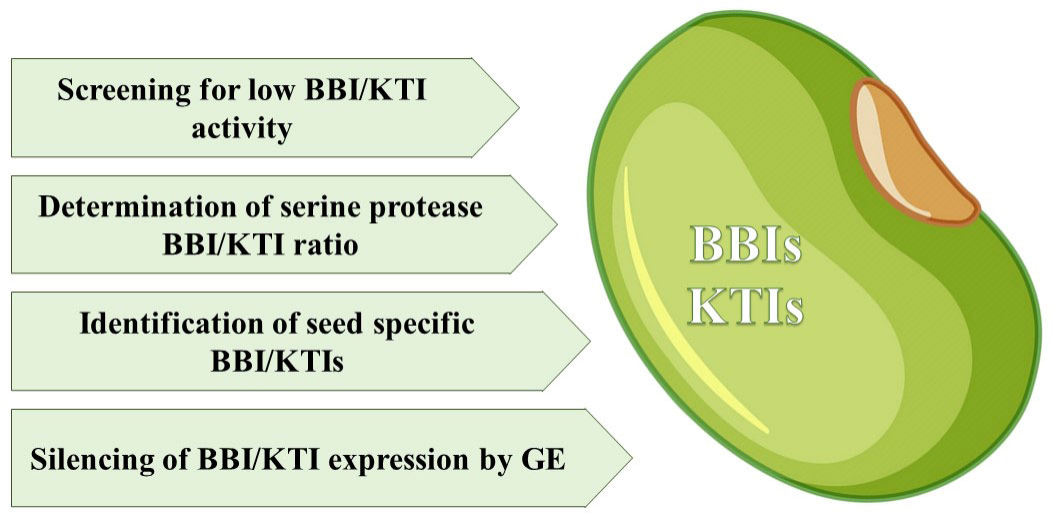
Actions to characterize and modify serine PIs in seeds.

Whether seed viability will be affected by lowering seed protease inhibitor levels, in order to achieve lower antinutrient activity, remains an interesting question. In general, current research on protease inhibitors is mainly focused on increasing, rather than decreasing, inhibitor activity to prevent, for example, protease activity of seed predators and seed pests as well as controlling endogenous seed proteases (Jamal et al., 2013; Grosse-Holz and van der Hoorn, 2016). As a first step screening of existing germplasm of beans for example, naturally occurring, low levels of serine protease inhibitors in the seeds, would be an important research task (for an overview see **Figure 1**). A high throughput and accurate HPLC-based method to determine TIr content is seeds has been developed by Rosso et al. (2018). The relationship between inhibitor amounts and seed viability also needs to be established. In this regard, we have already started to screen part of a European common bean core collection specifically for lower SPI activity in seeds to determine if their viability is depending on the SPI activity. Interesting would also be investigating in much more detail the existence of specific seed inhibitor genes essential for the development of viable seeds.

Another important aspect to be investigated in the future will be to evaluate the tolerance of low antinutrient seeds towards seed predators and pathogens. This might be compromised due to lower SPI activity. A further interesting question is also if a lower protease inhibitor amount will correlate with a higher, or lower, serine protease activity in seeds. A higher protease activity might actually be detrimental for seeds when premature degradation of storage proteins is allowed during seed development. This might ultimately severely affect seed germination and growth of the seedling.

Finally, in addition to classical breeding methods, application of genetic modification technologies would be, a promising option in lowering the expression of specific serine proteases in seeds. In cases where seed specifically expressed SPIs have been identified, selectively silencing these, in order to reduce antinutrients in seeds, by genome editing (GE) to produce inhibitor mutants would be a possible future strategy. Silencing such individual seed SPIs would allow to unravel specific and unique functions in the development of viable seeds. Application of GE, such CRISPR/Cas9 and TALEN (Zhang et al., 2018), has resulted in the nutritional improvement of a tomato which has already been released to the market (Waltz, 2022). A first success applying GE for detection of a seed specific protease/protease inhibitors has been the recent detection of the aspartic protease nepenthesins, which has been exclusively identified by the GE technology in non-viable seeds (Escandón et al., 2022). An advantage of the GE technology is that mutations created by this technology are considered to be almost identical to spontaneous genetic mutations since mutation inducer, the edited foreign gene, can be completely eliminated from the final genome-edited hosts after causing the mutation. Recently Rosso et al. (2021) developed a cost effective and breeder-friendly KASP SNP genotyping assay linked to low KTI content in soybean. This system may be extended to other crops to develop low KTI lines.

## Data availability statement

The original contributions presented in the study are included in the article/supplementary material. Further inquiries can be directed to the corresponding author.

## Author contributions

JV and KK are the main author of the perspective article completing the writing of the first draft of relevant literature. All authors contributed to the article and approved the submitted version.
